# Moral Injury in Healthcare Workers: Preliminary Results from a French Nationwide Survey

**DOI:** 10.1192/j.eurpsy.2025.1570

**Published:** 2025-08-26

**Authors:** L. Boyer, A. Duclos, G. Fond, P.-M. Llorca

**Affiliations:** 1 AMU, Marseille; 2 HCL, Lyon; 3 CHU Clermont Ferrand, Clermont Ferrand, France

## Abstract

**Introduction:**

Healthcare workers (HCW) face a growing crisis marked by high burnout levels (40-60%) and a rising intent to leave the profession. Current interventions focus mainly on individual resilience but have limited collective impact. Emerging evidence suggests that “Moral Injury” (MI)—a deep discord between HCW values and their practice, often worsened by organizational and economic constraints—may play a key role in this crisis.

**Objectives:**

The primary aim of this study was to describe the manifestations of MI among HCW in France, with a healthcare system characterized by universal health coverage. Secondary objectives included the psychometric validation of the Moral Injury Inventory (MII) for HCW, analysis of organizational determinants of MI, and modeling of the underlying mechanisms of this phenomenon.

**Methods:**

This cross-sectional, observational study was conducted nationally via an online survey. Measures included the MII, the Moral Injury Events Scale (MIES), the PHQ-9 for depression, the GAD-7 for anxiety, the Maslach Burnout Inventory (MBI), and the EuroQol 5-Dimensions (EQ-5D) for quality of life. Item Response Theory (IRT) was used to test the MII, including assessments of construct validity, and reliability. Structural equation modeling (SEM) was used to explore complex interactions between MI and other variables, including burnout, depression, and anxiety.

**Results:**

Nearly 3,000 HCW participated in the survey. Psychometric analysis confirmed that the MII met IRT model assumptions, including unidimensionality, local independence, and monotonicity, with fit indices indicating adequate model fit (RMSEA ≤ 0.08, CFI, TLI ≥ 0.95, and infit mean square statistic ranging between 0.7 and 1.0). Differential Item Functioning analysis revealed no item biases. Over 50% of HCW reported experiencing MI, with notable variability across structural/organizational characteristics. MI and burnout emerged as distinct yet frequently associated constructs, with moderate to strong correlations observed between them. Their combined presence had a cumulative negative impact on health outcomes, with affected HCW showing higher levels of depression, anxiety.

**Conclusions:**

This study highlights the high prevalence of MI among HCW in France, even within a robust healthcare system with universal coverage, and confirms that MI is distinct from burnout. These findings underscore the need for systemic interventions that address organizational factors, beyond the current individual-focused approaches used for burnout. Such a shift in focus would also help move away from the current tendency to over-burden and even blame HCW for systemic issues beyond their control. Given the global nature of the healthcare crisis, an international study is essential to identify systemic factors across healthcare settings, paving the way for a holistic, worldwide approach to supporting the healthcare workforce.

**Disclosure of Interest:**

None Declared

